# Effects of Crop Rotation Diversification and Livestock Integration on Above‐Ground Arthropod Dynamics Under Conservation Agriculture

**DOI:** 10.1002/ece3.71788

**Published:** 2025-07-21

**Authors:** Amandrie Louw, Johann Strauss, Pia Addison

**Affiliations:** ^1^ Department of Conservation Ecology and Entomology Faculty of AgriSciences, Stellenbosch University, Private Bag X1, Matieland Stellenbosch South Africa; ^2^ Western Cape Department of Agriculture Elsenburg South Africa

**Keywords:** canola, ground‐dwelling arthropods, plant‐dwelling arthropods, wheat

## Abstract

Diversification through integrating diverse crop species and livestock is key to enhancing above‐ground arthropod diversity and promoting the sustainability of cropping systems within conservation agriculture (CA) in South Africa. This study compared two crop rotation treatments, A (Wheat‐Wheat‐Wheat‐Wheat) and G (Canola‐Medics‐Wheat‐Medics), as part of a long‐term CA trial conducted in a wheat‐producing region of South Africa. For crop rotation system G, each phase of the rotation sequence was represented in separate plots annually. Surface‐dwelling arthropods were sampled using pitfall traps, while plant‐dwelling arthropods were captured through sweep‐net sampling. These methods comprehensively assessed above‐ground arthropod diversity 90 days after crop emergence. The results showed that crop rotation diversification positively influenced arthropod diversity, though the effects varied depending on the arthropod group and sampling position. Ground‐dwelling arthropods exhibited less pronounced differences between crop rotation systems, whereas plant‐dwelling arthropods displayed more crop‐specific variations. Analysis of individual arthropod orders revealed that the effects of crop rotation diversification varied across different arthropod groups. Our findings emphasize the importance of examining multiple arthropod groups to understand diversification's impacts fully within CA farming systems.

## Graphical abstract

Diverse crops in conservation agriculture systems can support a variety of arthropods, while sampling methods affected arthropod diversity outcomes. Canola‐Medics‐Wheat‐Medics rotation had a varied effect on arthropod diversity. We suggest that multiple taxa are necessary to compare diversification practices.

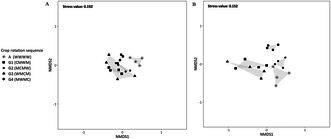

**Abbreviations**
ANOSIM: one‐way analysis of similarity
C: canola
CA: conservation agriculture
LSD: least significant difference
M: annual medics
NMDS: non‐metric multidimensional scaling
W: wheat

## 1 Introduction

Conservation agriculture (CA) is a farming system defined by three principles, including reducing soil disturbance, maintaining permanent soil cover with crop residues or live mulches, and encouraging diversification through crop rotations (Strauss et al. 2021). Together, these principles support sustainable practices, especially in the wheat production regions of the Western Cape province, South Africa. As these regions are often challenged with low and variable rainfall, alongside soil health challenges (Van Antwerpen et al. 2021). Considering that the integration of livestock has not only been found to further support diversification but also to support soil fertility, crop yields, and economic returns (Hilimire 2011; MacLaren et al. 2022; Petersen et al. 2007; Sekaran et al. 2021). For many smallholder farmers in the Global South, these economic benefits derived from livestock integration are particularly important due to the challenges posed by climate variability (Soussana and Lemaire 2013). Despite these advantages, there remains a growing need for these agricultural systems to further support productivity and actively promote biodiversity. This dual focus on economic viability and ecological health serves as an essential goal in further establishing resilient and sustainable agricultural landscapes within these regions.

Several studies have shown higher numbers of arthropod diversity and abundance in conservation agriculture (CA) systems (Mhlanga et al. 2020; Kadango et al. 2023). The impact of CA principles on arthropods is variable and dependent on several factors such as practices employed, local environmental conditions, and the duration from when CA principles were implemented (Conti 2015; Kadango et al. 2023). Furthermore, the impact of livestock‐integrated systems within CA on above‐ground arthropod communities remains underexplored, leaving the ecological implications and potential benefits unclear (Jones et al. 2021). This is particularly relevant as different arthropods and functional groups have been found to respond differently to diversification practices (Yekwayo et al. 2018; Torma et al. 2022).

While research has found that grazing affects habitat quality for arthropods, potentially enhancing their abundance and diversity (Rickert et al. 2012; Torma et al. 2022), grazing has a variable effect on above‐ground arthropods, with these benefits directly dependent on whether these organisms were in the plant canopy or on the soil surface (Zhu et al. 2023). More specifically, Garcia et al. (2023) and Mamabolo et al. (2024) found that livestock‐integrated systems positively affected ground‐dwelling arthropods, such as beetles and spiders. Conversely, livestock has also been shown to negatively affect plant‐dwelling arthropods through habitat destruction or direct consumption (Garcia et al. 2023).

Further research is needed to explore the effects of diversification with crops and livestock within CA systems on above‐ground arthropod community dynamics. This study investigated the impact of two crop rotation systems, including a wheat monoculture and a crop rotation system where wheat is rotated with canola and legume pasture that integrates livestock, within a larger CA trial in South Africa. Given that the trial is located in one of the two main winter wheat production areas in South Africa (Theron et al. 2021), this highlights the importance of this study within this region. We hypothesize that (1) the higher crop diversity and livestock integration crop rotation system under CA supports a diverse range of arthropod groups (Insects and spiders) with greater abundance, richness, and diversity of arthropods compared to a monoculture that is managed under no‐tillage and (2) each phase of the crop rotation sequence (i.e., crop identity) plays an important role in supporting distinct families within arthropod communities, thereby promoting the sustainability of the entire cropping system. Given differences at the community level, identifying taxa at the family level was considered essential, as suggested by Menta and Remelli (2020).

## 2 Materials and Methods

### 2.1 Site Description

Data were collected from a long‐term crop rotation trial established in 1996 on Langgewens Research Farm (33.28355 S, 18.707803 E) (https://glten.org/experiments/11), located in the Swartland region in the Western Cape province in South Africa. The Swartland falls within a Mediterranean climate with a warm temperate climate and hot, dry summers with a mean temperature ranging from 12.2°C to 24°C. Due to that, 80% of its total yearly rainfall of 395 mm falls between April and September, which is only sufficient to sustain a single cropping season per year. Crops are sown during April or May (austral Autumn) and harvested in October or November (austral Spring) and between each cropping season, fields (still covered by residue) are left fallow.

### 2.2 Trial Management

Crop rotations A and G (see Table 1) were sampled for above‐ground arthropods as part of a larger crop rotation systems trial. Farming practices in crop rotations A and G have remained consistent over the long term, and the only change made was the stocking rate during the pasture phase, which increased from 0.56 ewes per hectare to 3.32 ewes per hectare in 2009 during the annual medic pasture phase.

**TABLE 1:** Full description of wheat monoculture (A) and crop rotation systems (G). Crop rotation systems are part of the long‐term rotation trial on the Langgewens Research Farm (Western Cape province). Each phase of the crop rotation sequence was sampled during the 2020 cropping season. Wheat monoculture (A) serves as the control.

	Year 1	Year 2	Year 3	Year 4	Abbreviation
A	Wheat (W)	Wheat (W)	Wheat (W)	Wheat (W)	WWWW
G1	Canola (C)	Annual Medics (M)^a^	Wheat (W)	Annual Medics (M)^a^	CMWM
G2	Annual Medics (M)^a^	Canola (C)	Annual Medics (M)^a^	Wheat (W)	MCMW
G3	Wheat (W)	Annual Medics (M)^a^	Canola (C)	Annual Medics (M)^a^	WMCM
G4	Annual Medics (M)^a^	Wheat (W)	Annual Medics (M)^a^	Canola (C)	MWMC

^a^ Crop rotation (G) is integrated with livestock during the pasture phase. The cash crops included within crop rotation sequences are wheat, canola, and pasture crops as annual medics.

Crop rotation A consists of wheat [ *Triticum aestivum* L.] monocultures, which are less common but still implemented in the winter rainfall region of South Africa and was used as the control. Compared to crop rotation G, wheat as the primary cash crop is alternated with canola [ *Brassica napus* L.] and annual self‐regenerating (self‐seeded) medics [*Medicago truncatula Gaertn*. and *M. polymorpha L*.]. The medics serve as forage crops and are grazed by sheep [ *Ovis aries* L.], which are moved onto annual medics in April or May once the crops have re‐established. During the cash crop phase, the annual medics are chemically sprayed off. Crop rotation systems are managed using reduced soil disturbance practices. Crops are planted using a zero‐tillage (seed drill with disc opener, no soil disturbance) implement at a row spacing of 254 mm. Overall, no soil disturbances occur during the annual medics phase as it is self‐seeded and was only re‐sown when seed banks were below the critical threshold.

**FIGURE 1:**
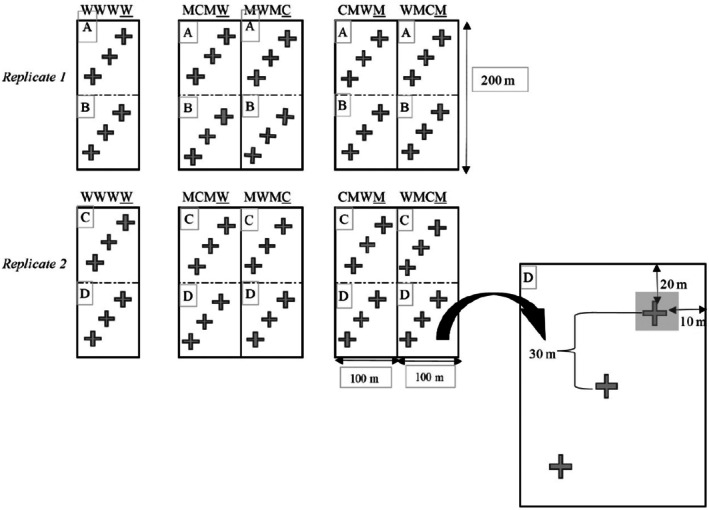
Overview sampling points within each crop rotation sequence of crop rotation treatments A (WWWW), G1 (CMWM), G2 (MCMW); G3 (WMCM), and G4 (MWMC) replicated twice within a randomised block design. In crop rotation (G), livestock is integrated during the pasture phase in G1(CMWM) and G3(WMCM).

The trial employs a repeated four‐year rotation, with each crop and pasture phase planted in separate 100‐by‐200‐m plots to ensure that all rotation phases are represented yearly. This ensures all crops are present annually, facilitating the analysis of year‐to‐year variation and reducing the influence of individual years on treatment effects. Each treatment (crop rotation systems A and G) is replicated twice within a randomised block design (Figure 1). Crop rotation A involves the same crop grown over 4 years, representing only one possible crop rotation sequence, while crop rotation G includes four possible crop sequence combinations (G1–4).

### 2.3 Sampling Above‐Ground Arthropods

Twelve sampling points were established per crop rotation sequence. Three sampling points (1–3) per sampling block (A) were arranged diagonally and consistently applied throughout data collection.

Above‐ground arthropods were sampled using two methods: sweep netting and pitfall traps. The sampling methods were selected to target associated arthropod groups with specific plant types within crop rotation sequences. More specifically, pitfall traps were used to collect surface‐dwelling arthropods, and sweep netting was used to sample plant‐dwelling arthropods. Sampling occurred in 2020 at 90 days after plant emergence (DAE), which corresponds to the flowering and seed‐filling phases for canola and wheat cash crops, respectively (Becker et al. 2020; Swanepoel and Labuschagne 2020).

#### 2.3.1 Ground‐Dwelling Arthropods

Pitfall traps were constructed using plastic jars of 250 mL with a 6 cm diameter and were sunk into holes in the ground prepared with a steel pipe and leveled to the soil ground (Majer 1978). Each trap was filled with 60 mL of pure propylene glycol and covered with a roof to prevent flooding (Cheli and Corley 2010; Montgomery et al. 2021). Propylene glycol was used as a non‐toxic preservative and was added to ensure no harm was caused when consumed by sheep.

Pitfall traps were left for 4–5 days, after which samples were collected, then cleaned using a 2 mm sieve with water and transferred to 80% ethanol for later identification in the laboratory (Magoba et al. 2015). Insects and spiders were sampled and grouped into morphospecies. Family‐level identification was made using a Zeiss stereo microscope (Stemi 305, Gottingen, Germany) with family identification keys as referred to (Goulet et al. 1993; Scholtz and Holm 2008; Whittington 2018). Spiders were identified by a taxonomic expert (Prof. C. R. Haddad from the University of the Free State).

#### 2.3.2 Plant‐Dwelling Arthropods

The sweep net used in this study had a diameter of 45 cm and a handle length of 75 cm. Sweep netting was conducted in the afternoons to allow plants to dry from morning dew or previous rain. Sampling consisted of walking at a standard pace (ca. 6 km/h), sweeping at every step, resulting in 10 sweeps per side and 40 sweeps around each sampling point (Swart et al. 2017). At the end of 40 sweeps, the net contents were emptied into a re‐sealable plastic bag and frozen at −20°C.

For each sample, arthropods were grouped into orders and morphospecies and then identified at the family level using a Zeiss stereo microscope (Stemi 305, Gottingen, Germany) and available keys (Goulet et al. 1993; Scholtz and Holm 2008; Whittington 2018). All specimens were stored in 80% ethanol. A single person conducted most of the sampling and sorting. The reference collection of arthropods collected in this study is stored in the Department of Conservation Ecology and Entomology, Stellenbosch University, South Africa.

### 2.4 Molecular Identifications

Molecular identifications were used for damaged Hemiptera and Lepidoptera specimens. All DNA extraction and sequencing were performed through Inqaba Biotechnical Industries (Pty) Ltd., Johannesburg, South Africa (https://inqababiotec.co.za/) and PCR amplification and sequencing were performed using primers C1‐J‐1718 (Simon et al. 1994) and C1‐N‐2776 (Hedin and Maddison 2001). Sequences were blasted in the Basic Local Alignment Search Tool (BLAST) (https://blast.ncbi.nlm.nih.gov/Blast.cgi) in the NCBI (https://www.ncbi.nlm.nih.gov/) database at the highest percentage identity to identify species.

### 2.5 Data Analyses

Community differences according to crop rotation sequences and abundance, richness, and diversity analyses were done at the morphospecies level, and community composition and sampling method specificity were used at family‐level identifications. All data analyses were performed using R statistics V4.3.3 (R CoreTeam 2022) and Statistica V14.0 (TIBCO Software Inc. 2020) with R integration middleware.

#### 2.5.1 Community Differences According to Crop Rotation Sequences

Differences in arthropod composition between crop rotation sequences were examined separately for ground and plant‐dwelling arthropods using non‐metric multidimensional scaling (NMDS) based on Bray–Curtis and Jaccard dissimilarity matrices in *vegan* (version 2.6–2) as described by Oksanen et al. (2017). This analysis is based on a distance matrix, allowing for more flexibility to analyze complex multi‐dimensional data in a small number of dimensions compared to other ordination techniques. A square root transformation was used on abundance data to reduce the relative differences among morphospecies groups.

This analysis was repeated using presence‐absence data to decrease the effect of abundant species and to ensure the outcomes of the analyses. Subsequently, cluster analysis was performed using group averaging, followed by a one‐way Analysis of Similarity (ANOSIM) to assess whether arthropod assemblages differ in composition (Clarke et al. 2014). Differences among crop rotation sequences and sampling methods were considered significant as the Global R statistic approached 1. Statistical significance was determined through permutation testing conducted using the *vegan* package, with *p*‐values less than 0.05 considered significant.

### 2.6 Abundance, Richness, and Diversity

To quantify differences between crop rotation sequences, (1) the abundance; (2) species richness, calculated as the total number of phenotypically distinct species; (3) diversity calculated with the Shannon‐Wiener diversity index (Shannon and Weaver 1949) and (4) the inverse of Simpson's index (Simpson 1949) to determine the sum of proportions of different species over the total abundance were determined. Both Shannon‐Wiener and inverse Simpson index were calculated using the diversity () function within the *vegan* package. For the differences in abundance, richness, and diversity according to the Shannon‐Wiener index and inverse Simpson's index, we used generalized linear mixed models (GLMM) using the glmmTMB package. We constructed models for each response variable. In these models, crop rotation, sampling method, and interaction between sequence and sampling method were set as fixed factors, and sampling block was set as a random variable within these models. The abundance data log was transformed and analyzed with a Gaussian distribution. A Poisson distribution for morphospecies richness and Shannon diversity index was used, and a gamma distribution (inverse link function) was used for the inverse Simpson's index. A pairwise comparisons test was done at a 5% significance level to evaluate differences between crop rotation sequences using the *emmeans* package (Searle et al. 1980). All models were tested for model fit using the DHARMa package. Differences were considered significant for *p*‐values less than 0.05.

#### 2.6.1 Arthropod Families Within Community Composition

Arthropod family‐level identifications were then used to study associations with crop rotation sequences using a two‐dimensional correspondence analysis across sampling methods within Statistica V14.0 (TIBCO Software Inc. 2020) with R integration middleware. Each arthropod order and the identified families were then used as column variables, and crop rotation sequences as the row variables. No supplementary row variables were used in either analysis.

#### 2.6.2 Sampling Method Specificity

Family‐level identification was used within each order and investigated with contingency tables and a maximum likelihood chi‐square test (not Pearson). Data were then visualized using a categorized histogram with family and sampling method to indicate which sampling method was more successful in collecting specific families.

## 3 Results

### 3.1 Community Differences According to Crop Rotation Sequences

The ANOSIM results indicate that the arthropod assemblages differed, and the crop rotation sequence had a significant effect when analyzing each of the sampling methods separately: ground‐dwelling (*R*: 0.468, *p*‐value < 0.05 (1e‐04)) and plant‐dwelling arthropods (*R*: 0.678, *p*‐value < 0.05 (1e‐04)). These results remained consistent with the presence and absence data (Figure 3A,B): ground (*R*: 0.6783, *p*‐value < 0.05 (1e‐04)) and plant‐dwelling arthropods (*R*: 0.4296, *p*‐value < 0.05 (1e‐04)).

**FIGURE 2:**
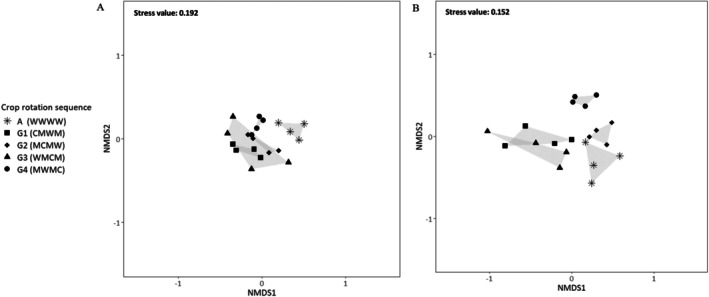
Non‐metric MDS ordination of crop rotation systems for each sampling method (ground‐dwelling and plant‐dwelling arthropods) based on averaged abundance data (square‐root transformed) per repetition: Figure (A) crop rotation sequences for ground‐dwelling arthropods. Figure (B) crop rotation sequences for plant‐dwelling arthropods. Crop rotation sequences are referred to in Table 1.

Moreover, Figure 2A,B, illustrate arthropod assemblages per sampling block (repetition) for each crop rotation sequence according to ground and plant‐dwelling arthropods. Dissimilarities were observed between the wheat monoculture A (WWWW) and G1 (CMWM), G2 (MCMW), G3 (WMCM), and G4 (MWMC) crop rotation sequences for ground‐dwelling arthropods. In contrast, for plant‐dwelling arthropods, the dissimilarities were between crop species wheat, medics, and canola, irrespective of the wheat monoculture A (WWWW) and the different stages of the crop rotation system G.

#### 3.1.1 Abundance, Richness, and Diversity According to Crop Rotation Sequences

The results indicated a significant interaction between crop rotation sequence and sampling method was observed for arthropod abundance, richness, and diversity. The total abundances per morphospecies (*p* < 1.43 × 10^−7^), morphospecies richness representing the total number of morphospecies (*p* < 0.009836), *and* average species richness were calculated using the Shannon diversity index (*p* < 0.0001129) and the inverse of Simpson's index (*p* < 1.73 × 10^−11^) (Figure 4A–D).

**FIGURE 3:**
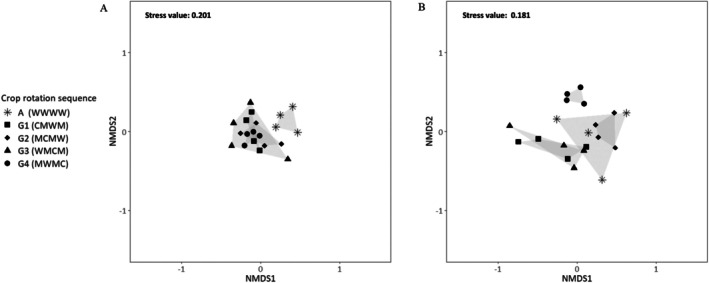
Non‐metric MDS ordination of crop rotation systems for each sampling method based on averaged presence‐absence abundance data (square‐root transformed) per repetition: Figure (A) crop rotation sequences for ground‐dwelling arthropods. Figure (B) crop rotation sequences for plant‐dwelling arthropods. Crop rotation sequences are referred to in Table 1.

**FIGURE 4:**
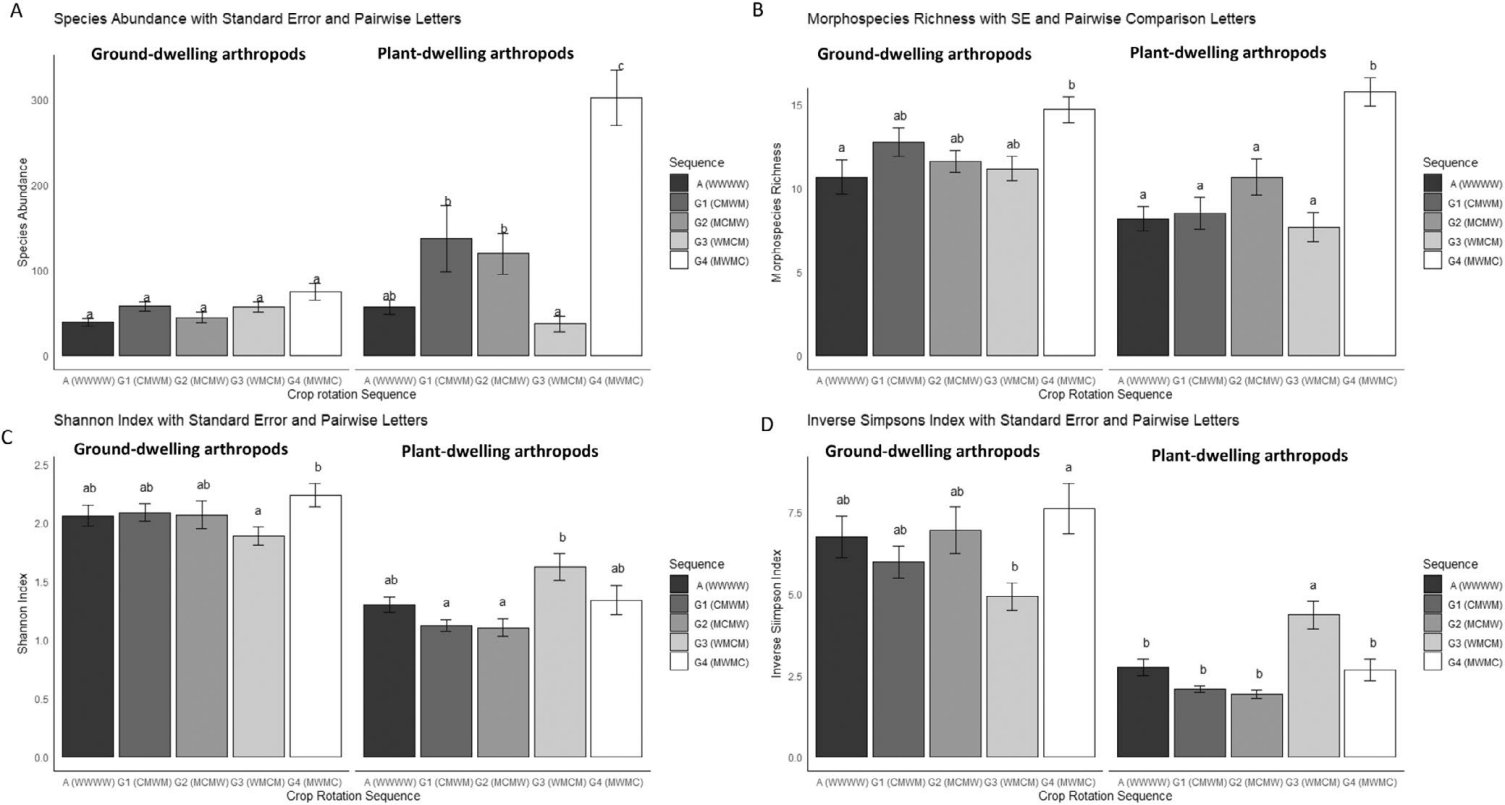
Species diversity in crop rotation sequences, as referred to in Table 1 Based on soil‐dwelling and plant‐dwelling arthropods. Bar graphs illustrate (A) the total average number of individuals; (B) morphospecies richness, which indicates the total average number of phenotypically distinct species; (C) average species richness assessed using the Shannon‐Wiener diversity index; and (D) the inverse of the Simpson's index.

Pairwise comparisons of crop rotation sequences indicated differences in diversity metrics for both soil‐dwelling and plant‐dwelling arthropods. Crop rotation sequence G4 (MWMC) differed significantly from wheat monoculture (WWWW) in abundance for plant‐dwelling arthropods (Figure 4A). Crop rotation sequence G4 (MWMC) also showed significant differences from wheat monoculture (WWWW) for both ground‐dwelling and plant‐dwelling arthropods in morphospecies richness (Figure 3B).

Further analysis of species diversity using the Shannon and inverse Simpson diversity indexes (Figure 4C,D) indicated significant differences between G3 (WMCM) and G4 (MWMC) for ground‐dwelling arthropods. For plant‐dwelling arthropods, significant differences between the same crop rotation sequences were observed using the inverse Simpson index. Plant‐dwelling arthropod crop rotation sequence G3 (WMCM) significantly differed from the wheat monoculture using the inverse Simpson's index.

### 3.2 Arthropod Families Within Community Composition

#### 3.2.1 Araneae

**FIGURE 5:**
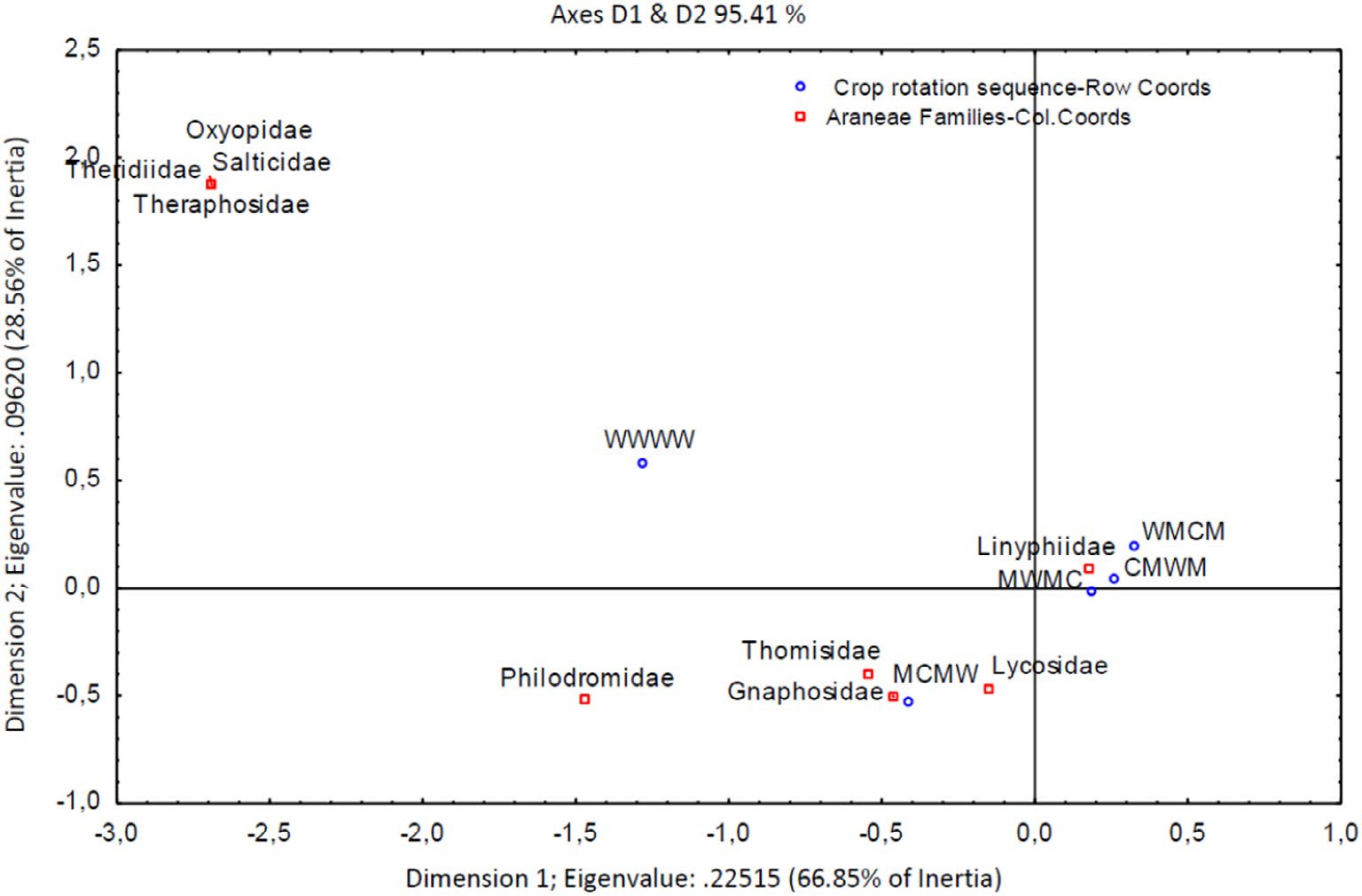
Two‐dimensional correspondence analysis showing the associations between crop rotation sequences and Araneae (spider) family groups. Crop rotation sequences are as follows: A (Wheat–Wheat–Wheat–Wheat); G1 (Canola–Annual Medics–Wheat–Annual Medics); G2 (Annual Medics–Canola–Annual Medics–Wheat); G3 (Wheat–Annual Medics–Canola–Annual Medics); and G4 (Annual Medics–Wheat–Annual Medics–Canola).

**FIGURE 6:**
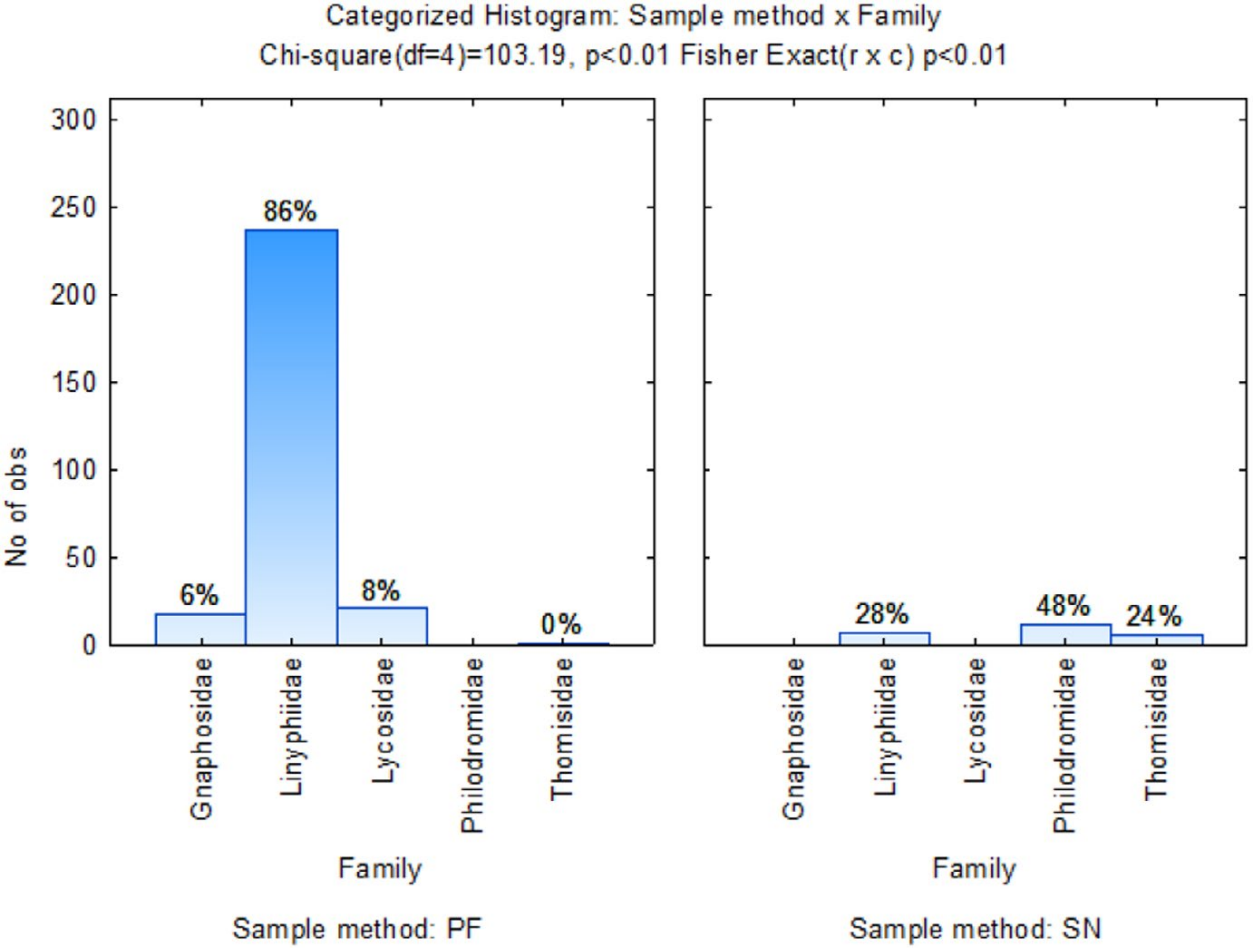
Categorized histogram with Araneae family's abundances in percentage (%) according to the pitfall (PF) and sweep‐net (SN) sampling method across crop rotation sequences.

Dimension 1 accounted for 66.85% of the variation in the graph (Figure 5), indicating a strong association between Linyphiidae spiders and G1 (CMWM), G2 (MCMW) and G3 (WMCM) crop rotation sequences as they are closely situated to each other on the positive side of the axis, at a high order of magnitude. Lycosidae, Thomsidae, Gnaphosidae, and Philodromidae were more closely associated with the crop rotation sequence G2 (MCMW). Oxyopidae, Salticidae, Theraphosidae, and Theudiidae were outliers with a high, positive order of magnitude in the top left quadrant and no associations with crop rotation sequences. No Araneae families showed any associations with the wheat monoculture. The highest number of Linyphiidae spiders (86%) was collected using the pitfall sampling method, while sweep net sampling only collected a maximum of 48% of samples, which were Philodromidae spiders (Figure 6).

#### 3.2.2 Coleoptera

**FIGURE 7:**
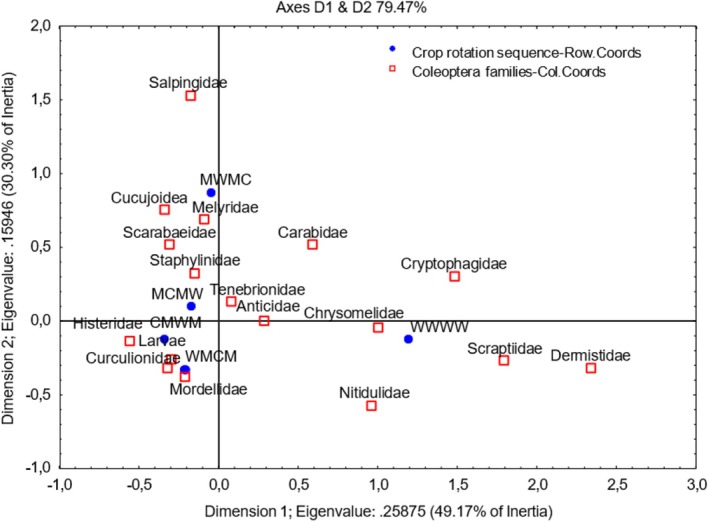
Two‐dimensional correspondence analysis illustrating the association between crop rotation sequence and Coleoptera family groups. Crop rotation sequences are as follows: A (Wheat–Wheat–Wheat–Wheat); G1 (Canola–Annual Medics–Wheat–Annual Medics); G2 (Annual Medics–Canola–Annual Medics–Wheat); G3 (Wheat–Annual Medics–Canola–Annual Medics); and G4 (Annual Medics–Wheat–Annual Medics–Canola).

**FIGURE 8:**
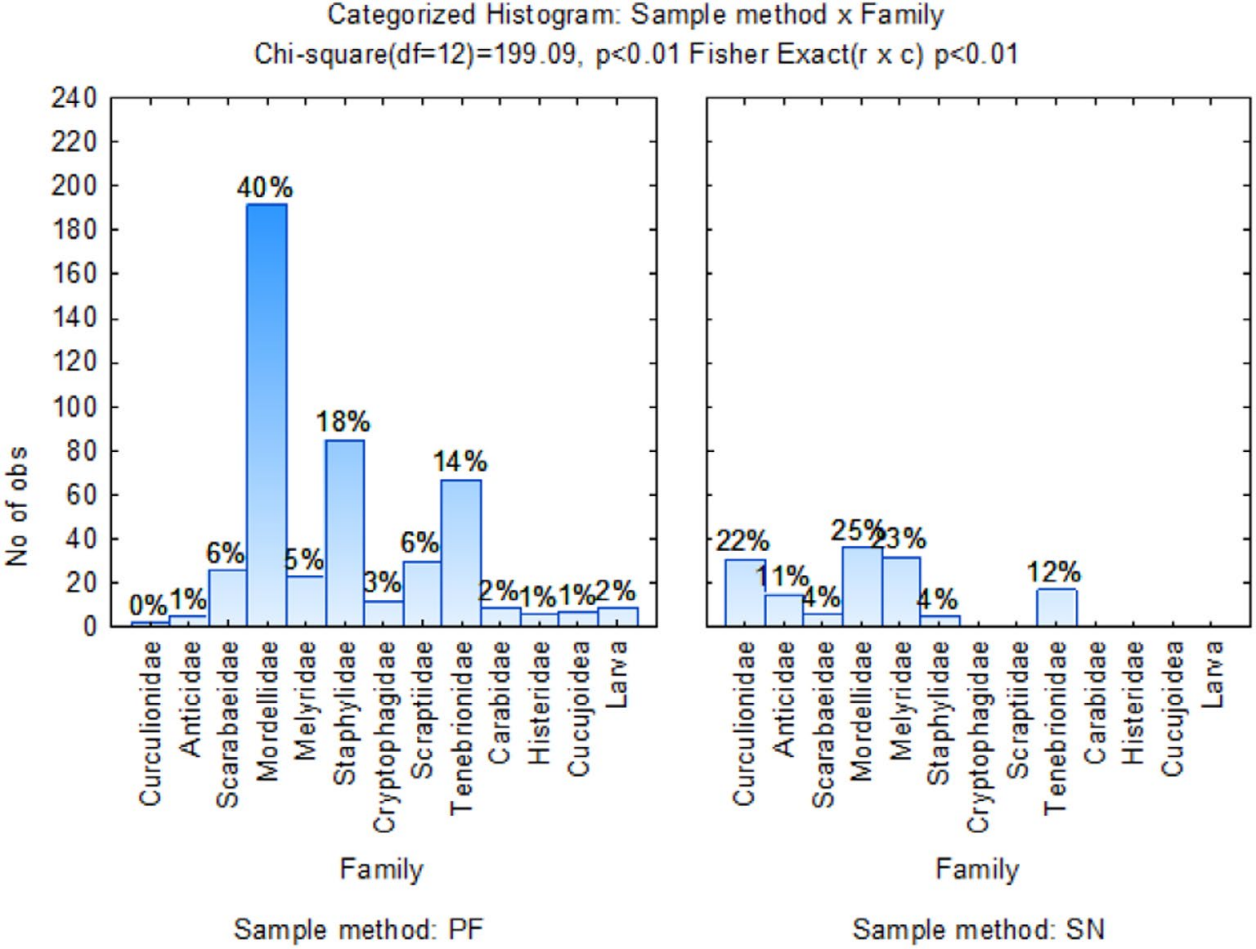
Categorized histogram with Coleoptera family's abundances in percentage (%) according to the pitfall (PF) and sweep‐net (SN) sampling method across crop rotation sequences.

Dimension 1 accounted for 49.17% of the variation in the graph (Figure 7), indicating a strong association between the wheat monoculture and Cryptophagidae, Chrysomelidae, Scraptiidae, Dermestidae, and Nitidulidae Coleoptera families. The other Coleoptera families are strongly correlated with crop rotation G1 (CMWM), G2 (MCMW), G3 (WMCM), and G4 (MWMC) crop rotation systems, except for Salpingidae, which appears as an outlier located in the high, positive order of magnitude in the top left quadrant. According to Figure 8, pitfall sampling was more successful than a sweep net for collecting Mordellidae beetles, collecting 40% and 25%, respectively.

#### 3.2.3 Diptera

**FIGURE 9:**
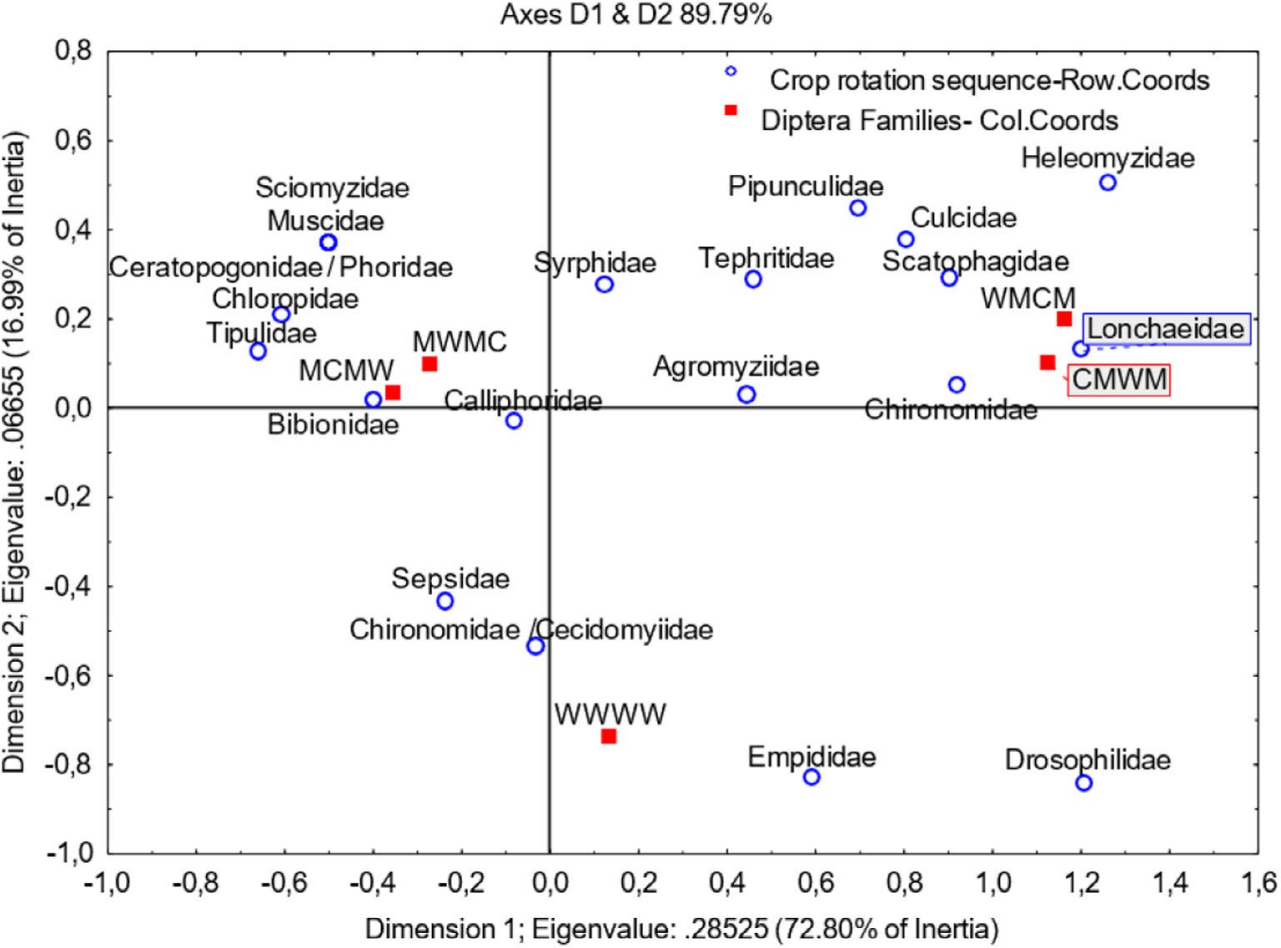
Two‐dimensional correspondence analysis illustrating the association between crop rotation sequence and Diptera family groups. Crop rotation sequences are as follows: A (Wheat–Wheat–Wheat–Wheat); G1 (Canola–Annual Medics–Wheat–Annual Medics); G2 (Annual Medics–Canola–Annual Medics–Wheat); G3 (Wheat–Annual Medics–Canola–Annual Medics); and G4 (Annual Medics–Wheat–Annual Medics–Canola).

**FIGURE 10:**
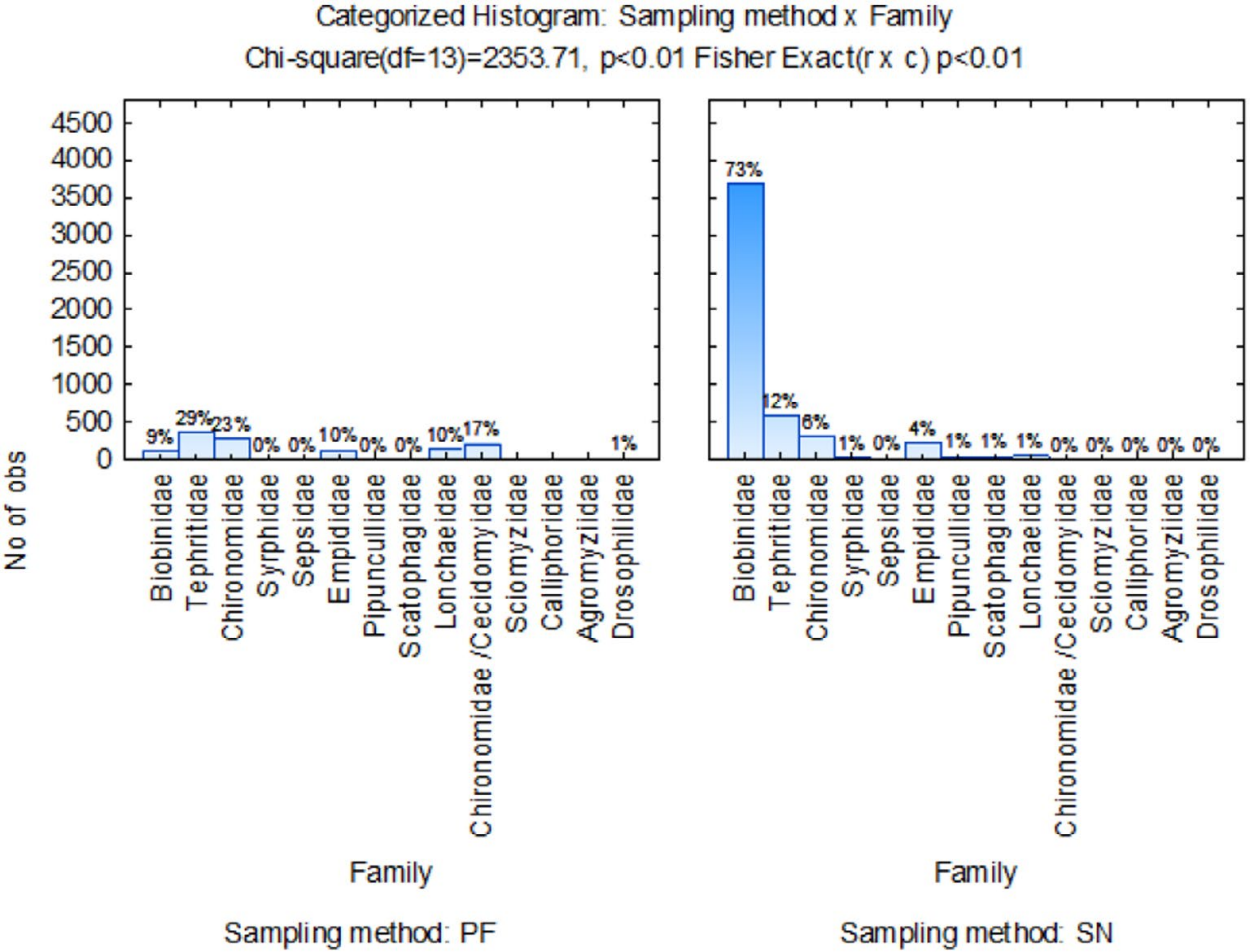
Categorized histogram with Diptera family's abundances in percentage (%) according to the pitfall (PF) and sweep‐net (SN) sampling method, across crop rotation sequences.

Dimension 1 accounts for 72.80% of the inertia variation (Figure 9). Dimension 2 (whilst only capturing 16.99% of the inertia) showed that Sciomyzidae, Muscidae, Ceratopogonidae/Phoridae, Chloropidae, Tipulidae, Bibionidae, and Calliphoridae were more closely associated with G2 (MCMW) and G4 (MWMC) crop rotation systems. Both crop rotation sequences that ended with medics were associated more with Chironomidae, Scatophagidae, Culicidae, and Heleomyzidae. As indicated in the lower left and right quadrants, Sepsidae, Chironomidae/Cecidomyiidae, and Empididae Diptera families were more closely associated with wheat monoculture. As shown in Figure 10, the sweep‐net sampling method collected the highest number of flies in the family Bibionidae. In contrast, pitfall trap sampling collected a maximum of 29% of samples in the Tephritidae family.

#### 3.2.4 Hemiptera

**FIGURE 11:**
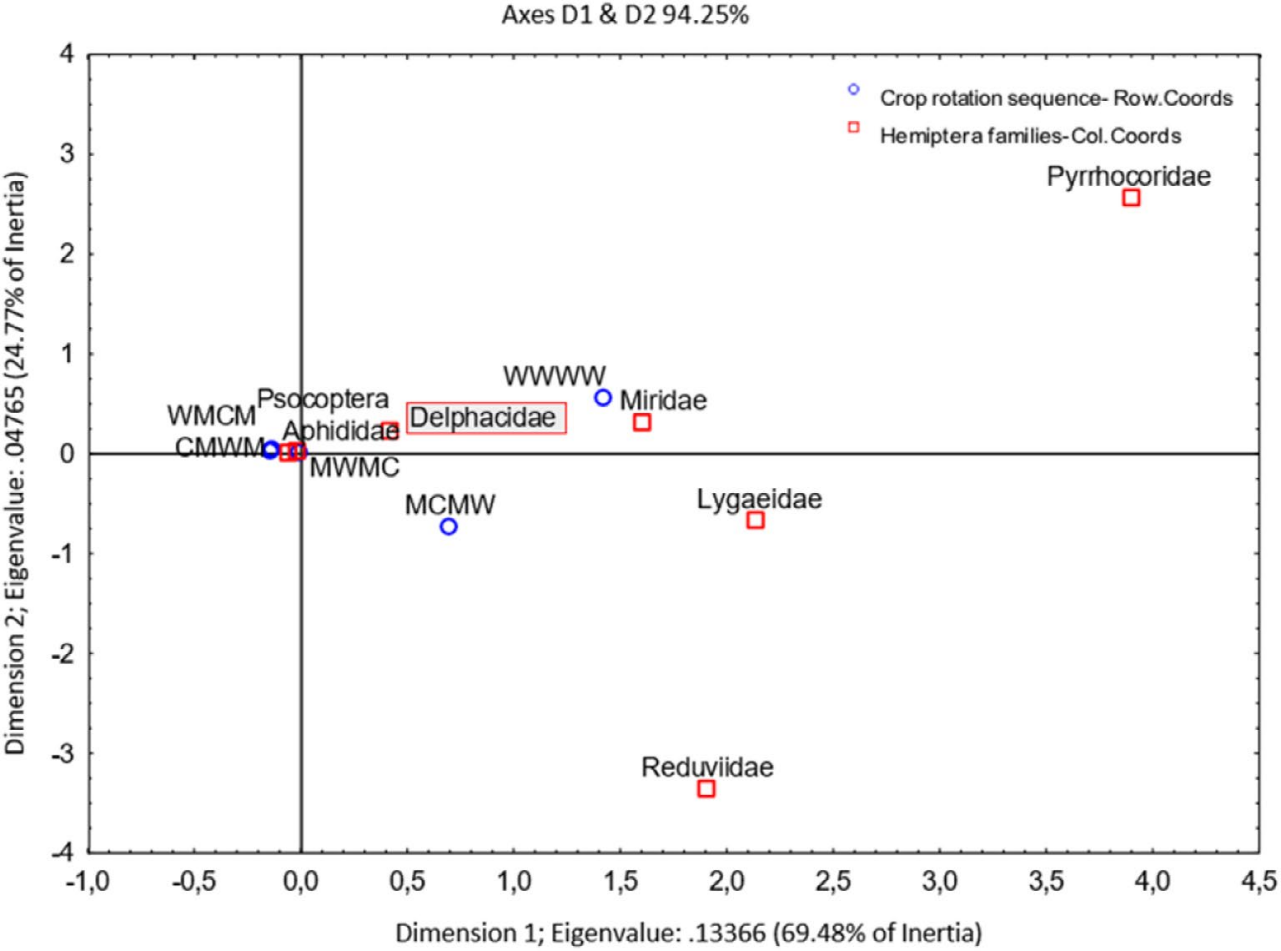
Two‐dimensional correspondence analysis illustrating the association between crop rotation sequence and Hemiptera family groups. Crop rotation sequences are as follows: A (Wheat–Wheat–Wheat–Wheat); G1 (Canola–Annual Medics–Wheat–Annual Medics); G2 (Annual Medics–Canola–Annual Medics–Wheat); G3 (Wheat–Annual Medics–Canola–Annual Medics); and G4 (Annual Medics–Wheat–Annual Medics–Canola).

**FIGURE 12:**
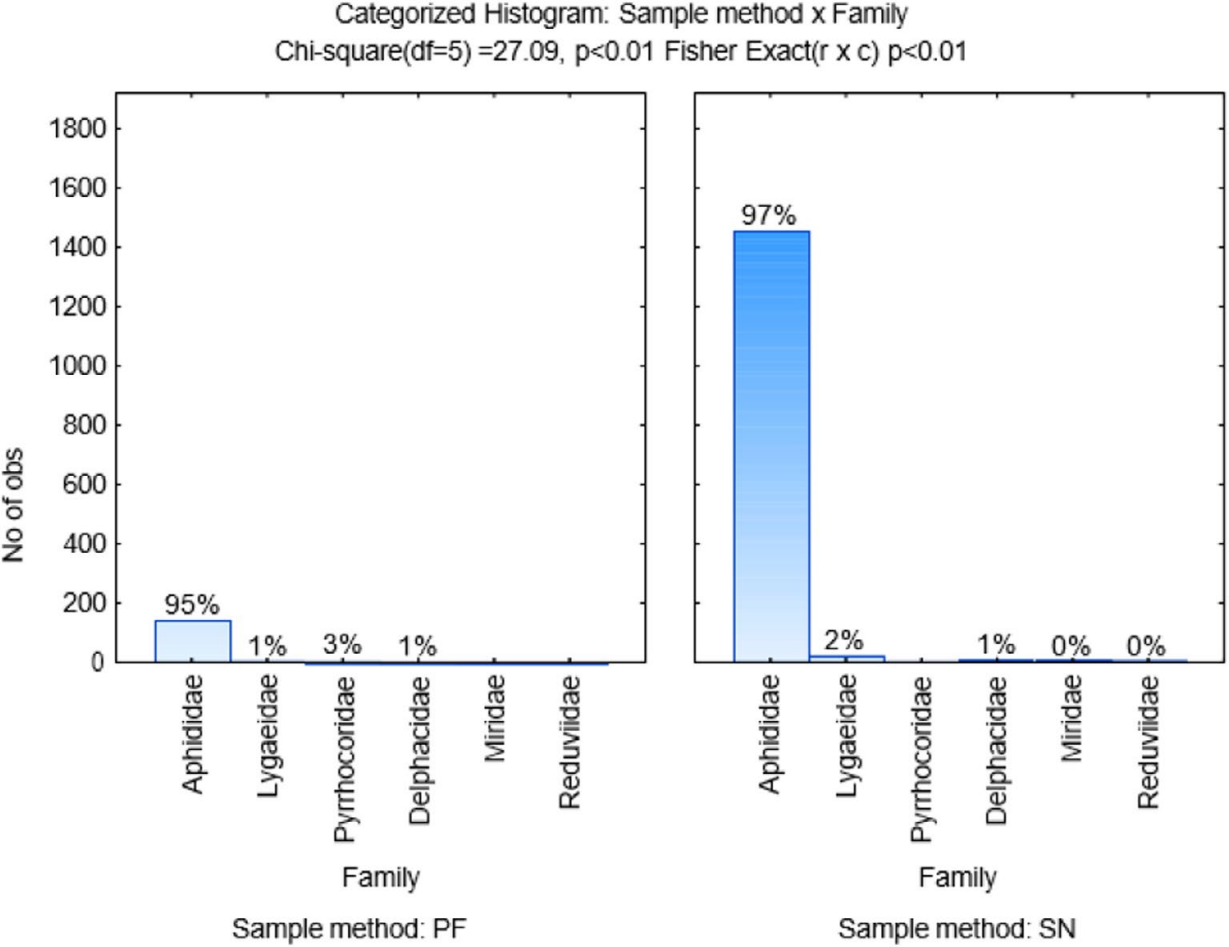
Categorized histogram with Hemiptera family's abundances in percentage (%) according to the pitfall (PF) and sweep‐net (SN) sampling method across crop rotation sequences.

Dimension 1 accounts for 69.48% of the variation, and Dimension 2 accounts for 24.77% of the variation in the graph (Figure 11). Crop rotation sequences that end with medics had a strong association with Aphididae. Furthermore, wheat monoculture is associated with Miridae, Lygaeidae, and Delphacidae. However, these families are also appreciably associated with crop rotation sequence G2 (MCMW). Further, Pyrrhocoridae appears to be an outlier with a high, positive order of magnitude in the top right quadrant, not associated with crop rotation sequences. Figure 12 indicates that over 90% of Hemiptera individuals caught were aphids, caught more or less equally using the pitfall (95%) and sweep net (97%) sampling methods. However, the number of observations for pitfall sampling (200) was far fewer than for sweep net sampling (1400).

#### 3.2.5 Hymenoptera

**FIGURE 13:**
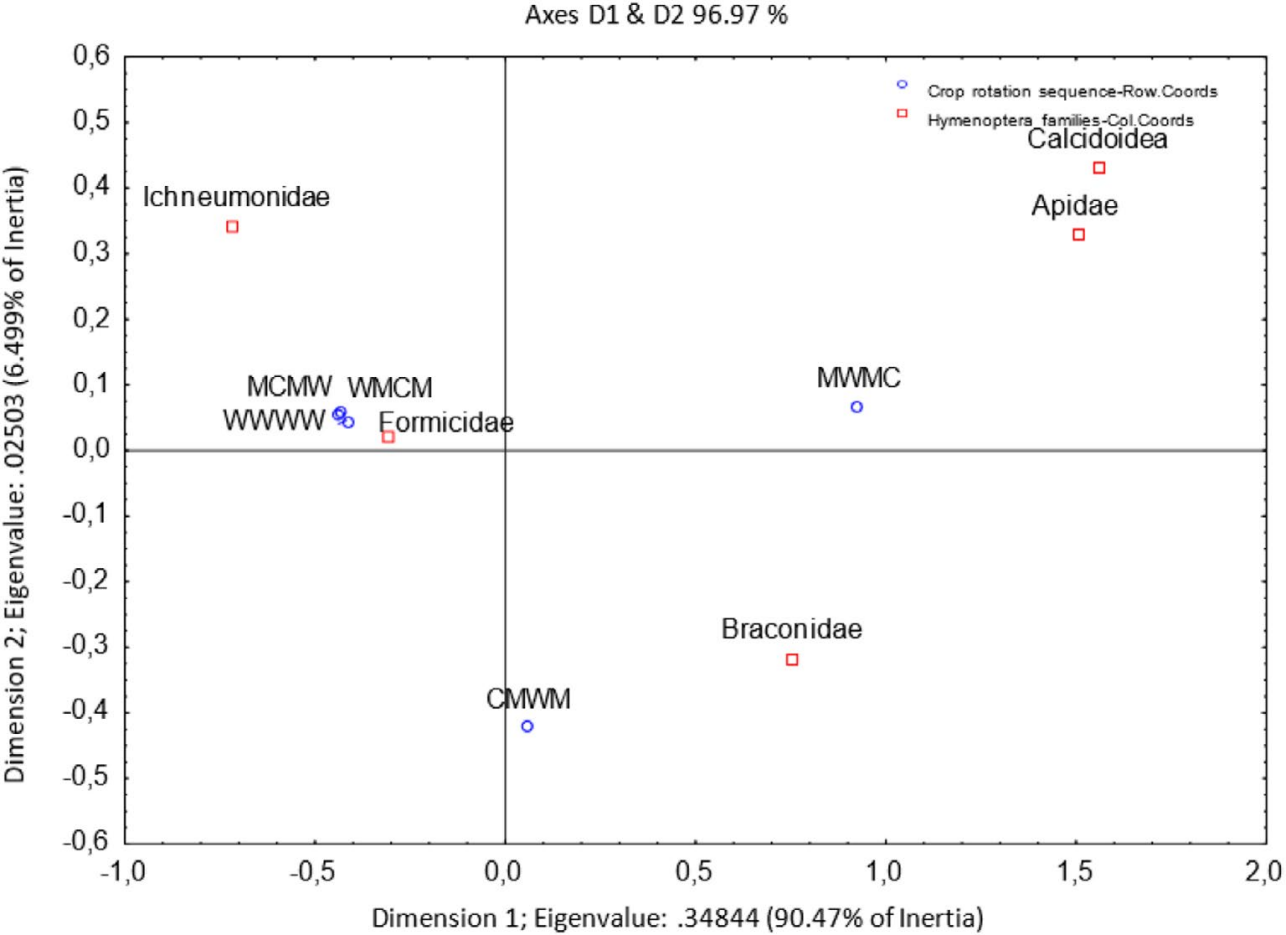
Two‐dimensional correspondence analysis illustrating the association between crop rotation sequence and Hymenoptera family groups. Crop rotation sequences are as follows: A (Wheat–Wheat–Wheat–Wheat); G1 (Canola–Annual Medics–Wheat–Annual Medics); G2 (Annual Medics–Canola–Annual Medics–Wheat); G3 (Wheat–Annual Medics–Canola–Annual Medics); and G4 (Annual Medics–Wheat–Annual Medics–Canola).

**FIGURE 14:**
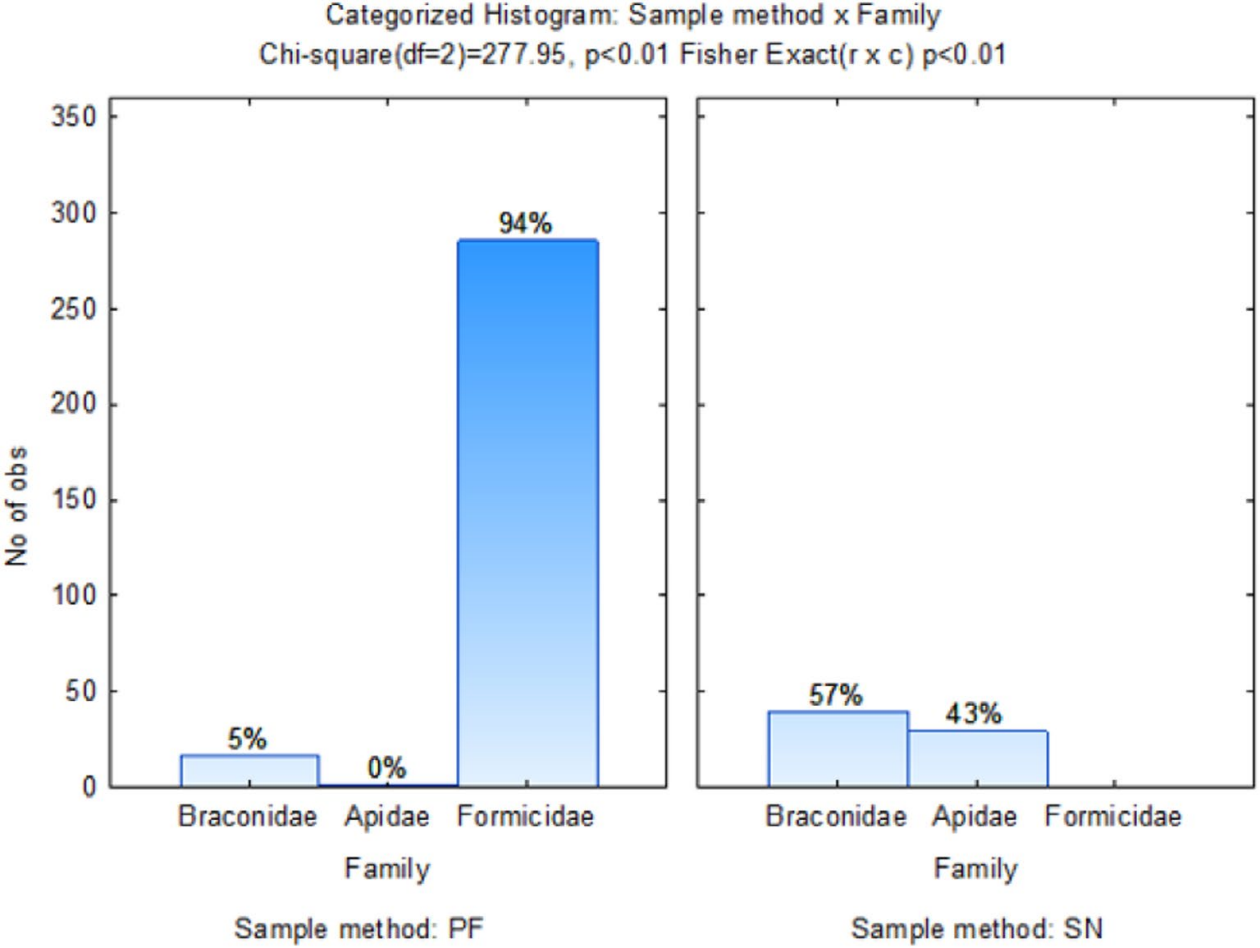
Categorized histogram with Hymenoptera family's abundances in percentage (%) according to the pitfall (PF) and sweep‐net (SN) sampling method across crop rotation sequences.

Dimension 1 accounts for 90.47% of the inertia, and Dimension 2 accounts for 6.49% of the inertia (Figure 13). The wheat monoculture (WWWW), G2 (MCMW), and G3 (WMCM) crop rotation sequences were more closely associated with Hymenoptera, such as Ichneumonidae and Formicidae families, as they were located in the left upper quadrant. Crop rotation sequence G4 (MWMC) appears more closely associated with the Apidae and Chalcidoidea families. G1 (CMWM) was more closely related to Braconidae families. As indicated in Figure 14, Formicidae was predominantly sampled using the pitfall sampling method.

#### 3.2.6 Lepidoptera

**FIGURE 15:**
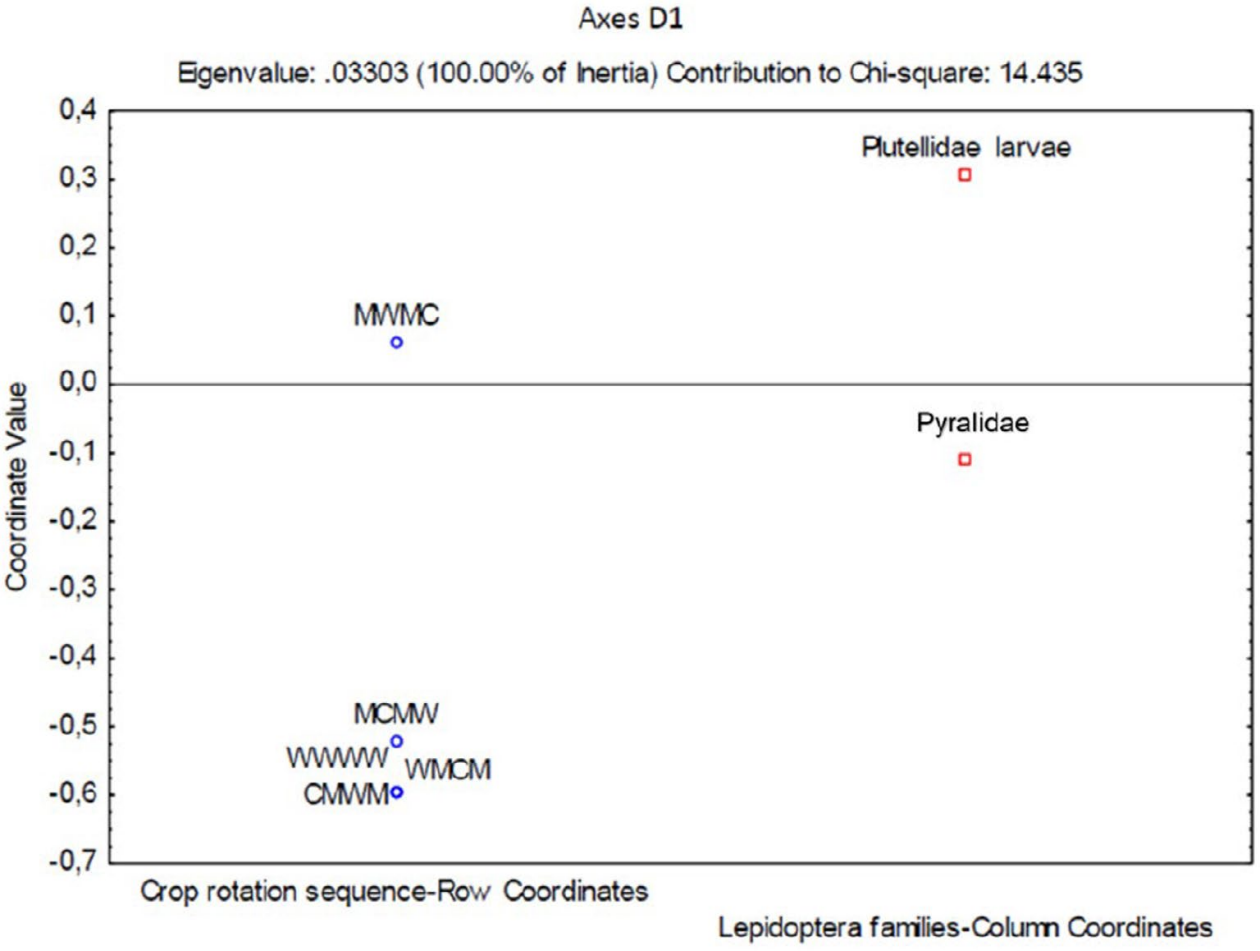
Two‐dimensional correspondence analysis illustrating the association between crop rotation sequence and Lepidoptera family groups. Crop rotation sequences are as follows: A (Wheat–Wheat–Wheat–Wheat); G1 (Canola–Annual Medics–Wheat–Annual Medics); G2 (Annual Medics–Canola–Annual Medics–Wheat); G3 (Wheat–Annual Medics–Canola–Annual Medics); and G4 (Annual Medics–Wheat–Annual Medics–Canola).

**FIGURE 16:**
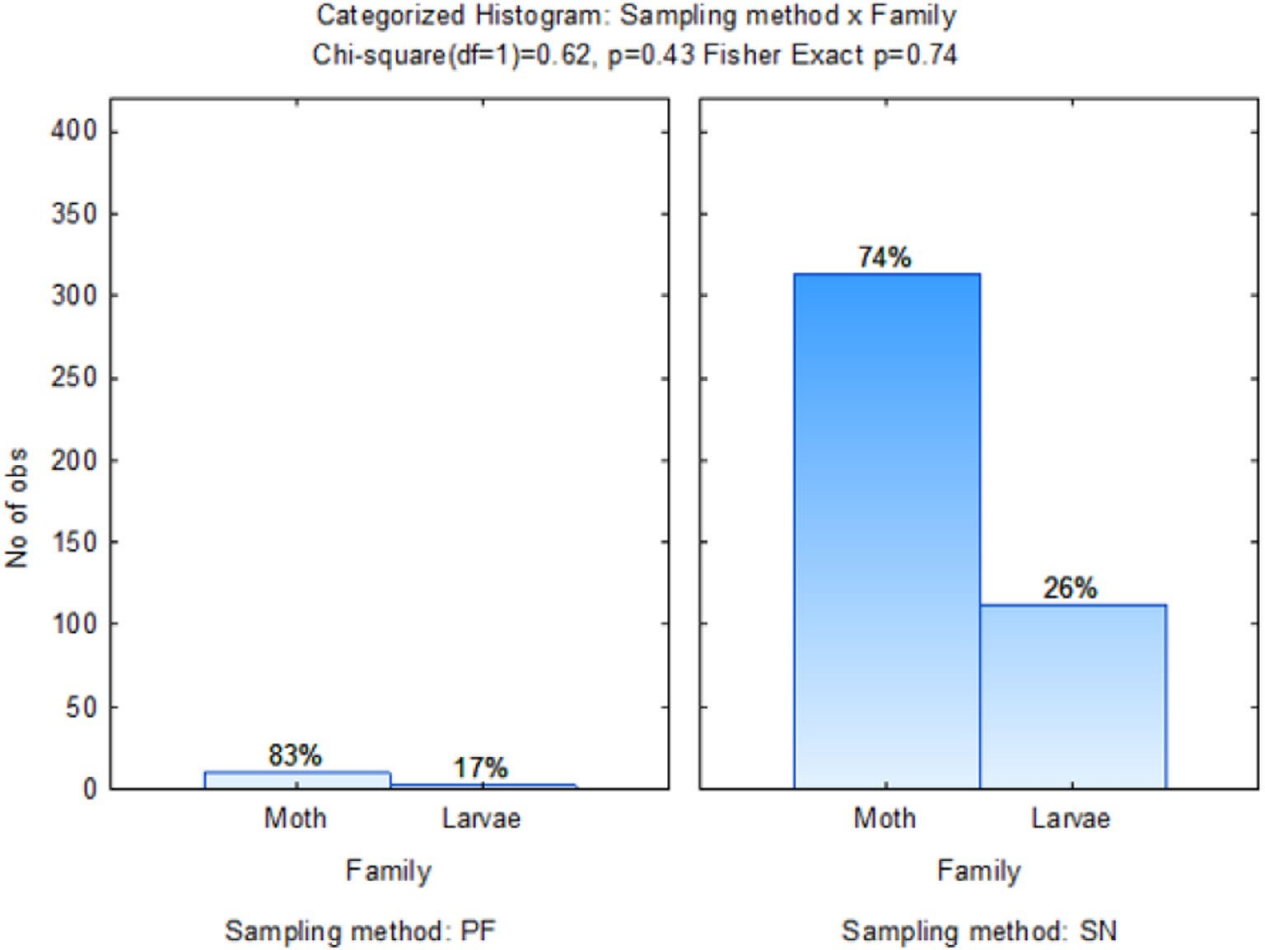
Categorized histogram with Lepidoptera family's abundances in percentage (%) according to the pitfall (PF) and sweep‐net (SN) sampling method across crop rotation sequences.

Figure 15 indicates the associations between Lepidoptera families comprising Plutellidae and Pyralidae with crop rotation sequences. Larvae were more closely associated with the MWMC crop rotation sequence. The adult moth samples were found between G4 (MWMC) and wheat monoculture, G1 (CMWM), G2 (MCMW), and G3 (WMCM) crop rotation sequences. The most moths (74%) were sampled using the sweep‐net sampling method (Figure 16).

## 4 Discussion

This study examined the effects of crop diversity and livestock integration under conservation agriculture (CA) practices on the above‐ground arthropod abundance, richness, diversity, and community composition. The results demonstrate that livestock‐integrated systems within the CA framework foster distinct arthropod communities, highlighting significant differences between ground‐dwelling and plant‐dwelling arthropods.

The non‐metric multidimensional scaling (nMDS) analysis revealed dissimilarities in arthropod community composition between crop rotation systems A and G. No differences in the abundance of ground‐dwelling arthropods were observed. Various studies suggest that soil disturbance has a more significant impact on ground‐dwelling arthropods compared to crop diversity, which may further explain the lack of differences in arthropod abundances found between crop rotation systems (Dassou et al. 2024; Jasrotia et al. 2023; Patterson et al. 2019).

The community in plant‐dwelling arthropods was crop specific, aligning with previous studies on the impact of crop identity on above‐ground arthropods (Mhlanga et al. 2020; Meyer et al. 2019; Patterson et al. 2019). Interestingly, only the G4 (MWMC) of crop rotation G showed greater arthropod abundance, richness, and diversity when compared to wheat monoculture. This demonstrated that diversification through crop rotation, especially with the incorporation of mass‐flowering crops such as canola ( *Brassica napus* ), can lead to increased arthropod abundance, richness, and diversity in crop rotation systems (Brandmeier et al. 2023).

The correspondence analysis revealed that each phase in the crop rotation was correlated with specific arthropod families within the community composition. The arthropods that responded most strongly to crop diversity were the Araneae (spiders) and the Coleoptera (beetles). The majority of the spider and beetle families were linked to the crop rotation system G. Surprisingly, non‐Araneae families were associated with the wheat monoculture (WWWW). This result is consistent with Meyer et al. (2019) and Berisha and Geci (2023), which revealed that the spider community structure was more positively associated with crops that provided complex vegetative structures. This is consistent with Berisha and Geci (2023) findings, which concluded that moderate grazing was positively correlated with spider families such as Linyphiidae and Thomisidae. Due to the fact that these spiders utilize complex plant structures for hunting. However, only Coleoptera families, such as herbivores (Chrysomelidae), fungivores (Nitidulidae), and saprophages (Scraptiidae and Dermestidae), were found to be correlated with wheat monoculture. This is likely caused by the fact that the wheat monoculture was also managed under reduced soil disturbance practices. The lack of differences is supported by Lalonde et al. (2012) that found that crop diversity has a limited effect on beetle species composition.

In addition, as crop rotation system G could offer more specialist food resources to arthropods compared to crop rotation A. As several insect families strongly responded to available resources provided by the canola and pasture crop phases within the crop rotation G. For the G4 sequence (MWMC) had a positive correlation with a variety of pollen‐attracted arthropod families, including Scarabaeidae, Syrphidae, Bibionidae, Calliphoridae, and Apidae. This concurs with the findings of Mlynarek (2022) regarding the benefit of these mass‐flowing crops to support pollinators. Similarly, sequences G1 (CMWM) and G3 (WMCM) were characterised by dung‐dwelling insects (Histeridae, Scathophagidae, Formicidae), likely attracted by the manure inputs from the livestock (Torma et al. 2022; Schmid et al. 2024).

Although our data were collected over a single season, the long‐term implementation of the crop rotation systems, which have been maintained with relatively few changes since their establishment in 1996, could provide significant contextual value. Long‐term field trials are widely recognized for their capacity to reveal gradual ecological responses and the legacy effects of management practices. Overcoming several short‐term data limitations, the enduring stability of these systems further enhances the likelihood of observing differences among treatments and reflecting underlying ecological trends rather than short‐term variability. As described by Bahlai et al. (2021) and Lange et al. (2023), long‐term experimental frameworks can enrich the interpretation of single‐season observations by placing them within broader temporal dynamics.

In examining the overall results, significant differences were observed among the various crop rotation systems. However, these differences could not be directly attributed to the presence of livestock, as long‐term grazing effects could have an effect on plant community composition, which may have had a more pronounced impact on above‐ground arthropods (Van Klink et al. 2014). Furthermore, no‐tillage farming systems require several years to fully establish (Pittelkow et al. 2015). Therefore, incorporation of crops such as canola into the rotation sequence could substantially support the diversity of above‐ground arthropods, thereby enhancing the ecological benefits of the entire rotation system within high crop diversity and livestock‐integrated systems managed under CA principles.

## 5 Conclusions

The present study offers valuable insights into the arthropods found in conservation agriculture (CA) systems within the Swartland region of the Western Cape province. This study is the first effort to understand the prolonged effects (18 years) of CA farming practices that integrate livestock and their impact on various above‐ground arthropods. While these findings are promising, further research is necessary to fully elucidate the complex and multifaceted interrelationships of diversification practices within CA systems and their influence on arthropods. In addition, researchers should be encouraged to include multiple taxa within their studies to make reliable comparisons between diversification practices.

## Author Contributions

**Amandrie Louw:** conceptualization (equal), data curation (equal), formal analysis (lead), funding acquisition (lead), investigation (lead), methodology (equal), visualization (lead), writing – original draft (lead). **Johann Strauss:** funding acquisition (equal), methodology (supporting), project administration (lead), resources (lead), supervision (supporting), writing – review and editing (equal). **Pia Addison:** conceptualization (equal), methodology (lead), resources (equal), supervision (lead), writing – review and editing (lead).

## Conflicts of Interest

The authors declare no conflicts of interest.

## Data Availability

All the data supporting results in this manuscript will be openly available in Zenodo at https://doi.org/10.5281/zenodo.13625508 upon acceptance for publication. https://zenodo.org/records/13625508?preview=1&token=eyJhbGciOiJIUzUxMiIsImlhdCI6MTc0NTk0MDk3NiwiZXhwIjoxNzY3MDUyNzk5fQ.eyJpZCI6IjFmNzFiYzJlLTY1ZmQtNDE1OC1hYWYwLTIzMGE3MDRjNjAzOCIsImRhdGEiOnt9LCJyYW5kb20iOiIzYjQ3ZjFkNWMyN2M5OGIxNTU1NjhlZmE2MTE5NjkyYSJ9.SngVhK‐G5nL‐Gm2px_AwM4ocUyH_5I9s9oDhv6D‐Q6sqzCyFi8L5B_jGX9yQaRf4dY0BwJ8‐oAsHn37v‐G1I3w.
